# Ultrasound reduces inflammation by modulating M1/M2 polarization of microglia through STAT1/STAT6/PPARγ signaling pathways

**DOI:** 10.1111/cns.14333

**Published:** 2023-07-03

**Authors:** Chin‐Hung Hsu, Yi‐Ju Pan, Yin‐Ting Zheng, Raymond Y. Lo, Feng‐Yi Yang

**Affiliations:** ^1^ Department of Biomedical Imaging and Radiological Sciences, School of Biomedical Science and Engineering National Yang Ming Chiao Tung University Taipei Taiwan; ^2^ Department of Psychiatry Far Eastern Memorial Hospital New Taipei Taiwan; ^3^ Department of Chemical Engineering and Materials Science Yuan Ze University Taoyuan Taiwan; ^4^ Department of Neurology Buddhist Tzu Chi General Hospital and Tzu Chi University Hualien Taiwan

**Keywords:** M1/M2, microglia polarization, neuroinflammation, PPARγ, STAT, ultrasound

## Abstract

**Introduction:**

Activated microglia can be polarized to the pro‐inflammatory M1 phenotype and the anti‐inflammatory M2 phenotype. Low‐intensity pulsed ultrasound (LIPUS) can attenuate pro‐inflammatory responses in activated microglia.

**Objective:**

This study aimed to investigate the effects of LIPUS on M1/M2 polarization of microglial cells and the regulatory mechanisms associated with signaling pathways.

**Methods:**

BV‐2 microglial cells were stimulated by lipopolysaccharide (LPS) to an M1 phenotype or by interleukin‐4 (IL‐4) to an M2 phenotype. Some microglial cells were exposed to LIPUS, while others were not. M1/M2 marker mRNA and protein expression were measured using real‐time polymerase chain reaction and western blot, respectively. Immunofluorescence staining was performed to determine inducible nitric oxide synthase (iNOS)‐/arginase‐1 (Arg‐1)‐ and CD68‐/CD206‐positive cells.

**Results:**

LIPUS treatment significantly attenuated LPS‐induced increases in inflammatory markers (iNOS, tumor necrosis factor‐α, interleukin‐1β, and interleukin‐6) as well as the expression of cell surface markers (CD86 and CD68) of M1‐polarized microglia. In contrast, LIPUS treatment significantly enhanced the expression of M2‐related markers (Arg‐1, IL‐10, and Ym1) and membrane protein (CD206). LIPUS treatment prevented M1 polarization of microglia and enhanced or sustained M2 polarization by regulating M1/M2 polarization through the signal transducer and activator of transcription 1/STAT6/peroxisome proliferator‐activated receptor gamma pathways.

**Conclusions:**

Our findings suggest that LIPUS inhibits microglial polarization and switches microglia from the M1 to the M2 phenotype.

## Graphical abstract

Low‐intensity pulsed ultrasound (LIPUS) inhibited inflammatory responses in activated microglia by shifting them from the M1 phenotype to the M2 phenotype. LIPUS treatment prevented M1 polarization of microglia and enhanced or sustained M2 polarization by regulating M1/M2 polarization through the signal transducer and activator of transcription 1/STAT6/peroxisome proliferator‐activated receptor gamma pathways.

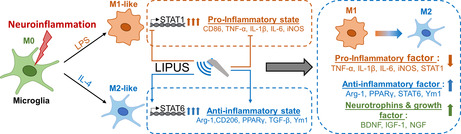

## 1 INTRODUCTION

Neuroinflammation is one of the major neuropathological characteristics of neurodegenerative diseases, such as Alzheimer's disease and Parkinson's disease.^1^, ^2^ Clinical failures in treating these diseases underline the obstacles to drug development, including delivery of a high enough drug concentration to the brain, because the blood–brain barrier (BBB) often prevents therapeutic levels from being achieved. Indeed, only a fraction of the drug that enters the brain reaches microglia. Therefore, the continuing lack of effective drugs to treat these diseases highlights the unmet need for developing innovative treatments.^3^, ^4^

A method of manipulating the microglial phenotype could potentially be an effective treatment for multiple human neurodegenerative diseases. Peroxisome proliferator‐activated receptor gamma (PPARγ) agonists have been shown to induce microglia to switch from M1 to M2 and reduce lipopolysaccharide (LPS)‐induced release of reactive oxygen species by inhibiting NF‐κB activity.^5^, ^6^, ^7^ Moreover, PPARγ activation has a protective effect by attenuating inflammation in the central nervous system (CNS). In previous studies, low‐intensity pulsed ultrasound (LIPUS) attenuated pro‐inflammatory responses as well as the decline in brain‐derived neurotrophic factor (BDNF) in LPS‐treated microglia.^8^ Moreover, LIPUS stimulation induced neuroprotection by inhibiting LPS‐induced activation of TLR4/NF‐κB inflammatory signaling and by enhancing the associated CREB/BDNF expression in LPS‐treated mice.^9^ Zhang et al. showed LIPUS induced the polarization change of inflammatory macrophages and resident macrophages in a rat model of spinal fusion.^10^ Nevertheless, whether LIPUS is involved in regulating microglial polarization and its molecular mechanisms is still unknown.

Microglia, the resident macrophages of the CNS, maintain the neural environment and respond to neural damage and repair by switching between different activation states.^11^ Inflammatory responses in the brain, which are indicated by changes in the properties of microglia, play a crucial role in multiple CNS diseases.^12^ Microglia are the first line of defense in the brain and have protective functions. They initiate an inflammatory response when a danger signal is detected. This may be triggered by microglial activation and consequent inducible nitric oxide synthase (iNOS) upregulation and secretion of several inflammatory cytokines.^13^ On the other hand, microglia can produce anti‐inflammatory cytokines, inhibit local inflammation, and promote trophic support by secreting neurotrophins.^14^ Therefore, microglia can be either cytotoxic or neuroprotective depending on their current phenotype. The shifting microglial phenotype has recently become a therapeutic strategy to treat multiple neurodegenerative diseases.^15^

Microglia can be polarized to the classically activated pro‐inflammatory M1 phenotype or the alternatively activated anti‐inflammatory M2 phenotype.^16^ M1 and M2 phenotypes are stimulated by LPS and interleukin‐4 (IL‐4), respectively.^17^ M1 microglia release high levels of pro‐inflammatory mediators, such as tumor necrosis factor‐α (TNF‐α), interleukin‐1β (IL‐1β), and interleukin‐6 (IL‐6), in addition to increased levels of oxidative metabolites, such as iNOS, and cell surface markers, CD86 and CD68.^18^, ^19^ These inflammatory factors are mediated by the signal transducer and activator of transcription 1 (STAT1) signaling pathway. In contrast, M2 microglia release beneficial mediators, including IL‐10, arginase‐1 (Arg‐1), Ym1, mannose receptor (CD206), transforming growth factor beta (TGF‐β), and several neurotrophic factors, such as BDNF, insulin‐like growth factor‐1 (IGF‐1), and nerve growth factor (NGF), which are mediated by the STAT6 signaling pathway.^16^, ^20^, ^21^ Furthermore, the M2 microglial phenotype can be categorized into M2a, M2b, and M2c subtypes, which can be distinguished by changes in expression of the relevant factors.^22^, ^23^ M1/M2 polarization of microglia can be regulated through STAT1 or STAT6 phosphorylation.^24^

Since LIPUS is a local treatment and is noninvasive, its targeted stimulation to activate microglia at the site of neuroinflammation could be highly beneficial. Therefore, this study aimed to investigate whether LIPUS has the ability to inhibit M1 polarization and promote M2 polarization and subsequently induce an M1‐to‐M2 phenotypic shift in microglia. The results of our study may have implications for treating inflammation‐related neurological diseases.

## 2 MATERIALS AND METHODS

### 2.1 Pulsed ultrasound system

LIPUS was generated by a therapeutic ultrasound generator (ME740, Mettler Electronics) and a 1‐MHz plane transducer (ME7410, Mettler Electronics; 9.3‐cm^2^ effective radiating area) with 2‐ms burst lengths at a 20% duty cycle and repetition frequency of 100 Hz. The spatial average intensity (I_SAI_) over the plane transducer head was 30 mW/cm^2^; it was measured with a radiation force balance (Precision Acoustics) in degassed water. The ultrasound parameters were selected based on the results of our previous study.^8^ The LIPUS was transmitted from the plane transducer to the bottom of the cell culture plate. Ultrasound transmission gel (Pharmaceutical Innovations) was used to cover the area between the transducer and the plate to maximize ultrasound transmission. Each microglial cell sample was treated with three episodes of LIPUS stimulation using triple sonication. To reduce the thermal effect of ultrasound, the duration of each sonication was 5 min, with a 5‐min interval between two sonications. Thus, the total sonication time of LIPUS stimulation was 15 min.

### 2.2 Cell cultures

BV‐2 cells, derived from primary mouse microglial cells, are the most frequently used substitute for primary microglia. There were no animal subjects in this study. The mouse BV‐2 microglial cell line was purchased from American Type Culture Collection (ATCC). The cells were grown on a six‐well plate (each well diameter = 34.8 mm) at an appropriate density in Dulbecco's Modified Eagle's Medium (Gibco) supplemented with 10% (v/v) heat‐inactivated fetal bovine serum, 100 μg/mL of streptomycin, and 100 U/mL of penicillin (Hyclone). They were maintained in a humidified 5% CO_2_/95% air environment at 37°C. The cells were incubated for a further 24 h in a humidified incubator. A cell density of 1 × 10^5^ cells/well was prepared for subsequent experiments. To promote polarization to the M1 or M2 phenotype, microglia were treated with LPS (10 ng/mL) or IL‐4 (20 ng/mL), respectively, for 8 h. BV2 cells were divided into six groups: control, LIPUS, LPS, LPS + LIPUS, IL‐4, and IL‐4 + LIPUS. The control group received no treatment except for a change of medium. The LIPUS group received the medium and LIPUS. The LPS group and LPS + LIPUS group consisted of microglia stimulated with LPS without and with LIPUS, respectively. The IL‐4 group and IL4 + LIPUS group consisted of microglia stimulated with IL‐4 without and with LIPUS, respectively. The LPS + LIPUS and IL‐4 + LIPUS groups were treated with LIPUS for a sonication time of 15 min 4 h after LPS and IL‐4 administration, respectively.

### 2.3 Cell viability measurements

The 3‐(4,5‐dimethylthiazol‐2‐yl)‐2,5‐diphenyltetrazolium bromide (MTT) assay was performed in this study to measure cell viability. BV2 microglia were incubated with PBS, LPS, or IL‐4 for 4 h and stimulated with/without LIPUS. Then, the cells were treated with MTT for 4 h and the formazan formed was dissolved with dimethyl sulfoxide (1 mL/well). Optical density was determined at 570 nm using a spectrophotometer (TECAN Sunrise™).

### 2.4 Real‐time polymerase chain reaction

Total RNA was isolated from cells using an RNeasy Mini Kit (QIAGEN) according to the manufacturer's instructions. RNA concentration and purity were determined using NanoDrop ND‐1000 (Thermo Fisher Scientific) according to the manufacturer's instructions. Reverse transcription was performed using a ToolsQuant II Fast RT Kit (BioTools) according to the manufacturer's instructions. RT‐PCR assay was performed using SYBR (KAPA SYBR FAST qPCR Master Mix (2×), ABI Prism™) in StepOnePlus™ (Applied Biosystems), according to the manufacturer's instructions. Table S1 summarizes the primers used in this study. β‐actin was used to normalize mRNA in the cells. Each sample was analyzed in triplicate for yield validation. The 2^−ΔΔCt^ method was used to determine the relative quantification of gene expression levels.

### 2.5 Western blot analysis

The different groups of cells were harvested and lysed in lysis buffer (RIPA 98%, EDTA 1%, and protease and phosphatase inhibitor 1%; Thermo Fisher Scientific Inc.), followed by centrifugation at 12500 rpm for 30 min at 4°C. Protein concentrations were quantified with the dye‐based protein assay (Bio‐Rad Laboratories Inc.) according to the manufacturer's instructions. The cell lysates (35 μg) were separated by sodium dodecyl sulfate polyacrylamide gel electrophoresis on 12% gels and transferred to polyvinylidene difluoride membranes (0.22 μm; Bio‐RAD). The blots were blocked with 5.5% fat‐free dry milk in TBST (1 mM Tris, 150 mM NaCl, 0.1% Tween20, pH 7.4) RT for 1 h and probed with primary antibodies at 4°C overnight. TNF‐α, IL‐1β, α‐tubulin, iNOS, Arg‐1, p‐STAT1, STAT1, p‐STAT6, and STAT6 were the primary antibodies. After being washed with PBST buffer, signals were developed using Western Lightning ECL Pro reagent (Bio‐Rad). Western blot signals were analyzed and visualized using a biomolecular imager (ImageQuant™ LAS 4000, GE Healthcare Bio‐Sciences AB).

### 2.6 Immunofluorescence

BV2 cells were incubated with 10 ng/mL of LPS in humidified 5% CO_2_/95% air for 24 h at 37°C. The cells were fixed in 4% paraformaldehyde for 10 min at room temperature and washed three times with PBS. The cells were treated with 0.1% Triton X‐100 for 5 min at room temperature. The washed cells were incubated with a blocking serum for 1 h and then incubated overnight with primary antibody (α‐tubulin). The cells were then washed and incubated with secondary antibodies for 1 h in a dark room. For nuclear staining, the cells were incubated with 10 μL of DAPI for 10 min. The slide was finally washed and mounted for microscopic examination. The stained cells were detected by fluorescence microscopy. The number of cells positive for α‐tubulin was counted in a 0.302‐mm^2^ area in three non‐overlapping fields under 200× magnification. The mean signal intensities for iNOS, Arg‐1, CD68, and CD206 were quantified in a 0.302‐mm^2^ area in three non‐overlapping fields with Image‐Pro Plus software (Media Cybernetics) in a blinded manner.

### 2.7 Statistical analysis

All data are shown as mean ± standard error of the mean. The Shapiro–Wilk test was used to test for normality. One‐way ANOVA, followed by Tukey's post hoc test, was used to determine differences between groups. The level of statistical significance was set at *p* < 0.05.

## 3 RESULTS

### 3.1 Effects of LIPUS, LPS, and IL‐4 treatment on microglia

**FIGURE 1:**
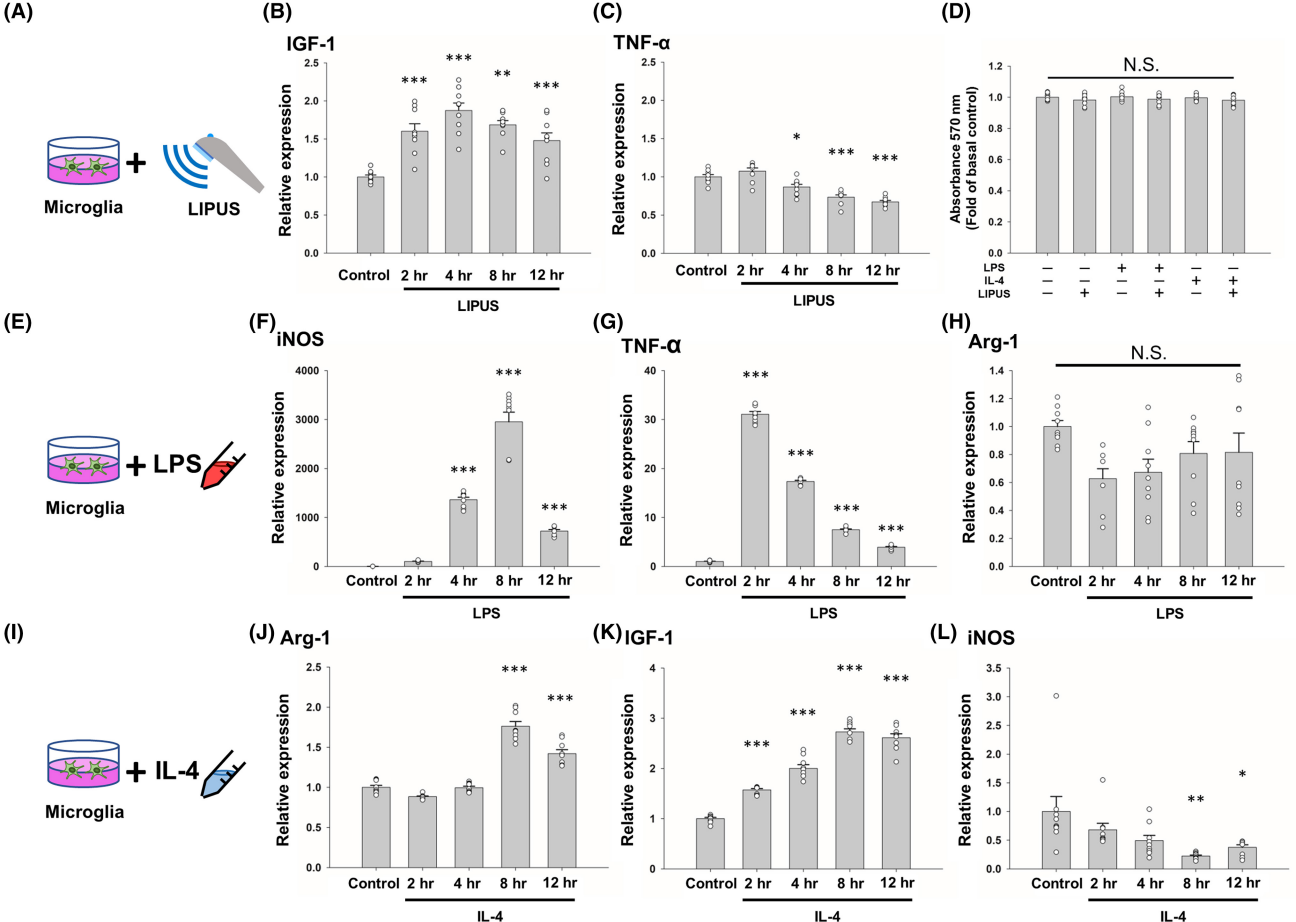
Effects of LIPUS, LPS, and IL‐4 treatment on the expression of M1‐ and M2‐related factors. (A) Microglial cells were treated with LIPUS. Cells were evaluated for (B) IGF‐1 and (C) TNF‐α mRNA expression at 2, 4, 8, and 12 h after LIPUS treatment. (D) No significant difference was found in the growth of microglia subjected to LIPUS, LPS, or IL‐4 treatment compared with the control group. (E) Microglial cells were treated with LPS and evaluated for (F) iNOS, (G) TNF‐α, and (H) Arg‐1 mRNA expression at 2, 4, 8, and 12 h after LPS treatment. (I) Microglial cells were treated with IL‐4 and evaluated for (J) Arg‐1, (K) IGF‐1, and (L) iNOS mRNA expression at 2, 4, 8, and 12 h after IL‐4 treatment. *denotes a significant difference between the control group and the other four groups. *p < 0.05; **p < 0.01; ***p < 0.001; N.S. = not significant; *n* = 9.

First, we examined the effect of LIPUS on microglia (Figure 1A) as it can increase the expression of neurotrophic factors in microglia.^8^ When exposed to LIPUS, microglia exhibited significant time‐dependent changes in IGF‐1 mRNA expression (*p* < 0.01; Figure 1B). The expression of IGF‐1 reached a maximum at 4 h after LIPUS stimulation (*p* < 0.001; Figure 1B). On the other hand, LIPUS significantly decreased TNF‐α mRNA expression 4 h after stimulation (*p* < 0.05; Figure 1C). LIPUS, LPS, IL‐4, or a combination of the three did not affect cell viability compared with the control cells (Figure 1D). Second, we examined the effect of LPS on microglia (Figure 1E). iNOS and TNF‐α mRNA expression showed time‐dependent changes in LPS‐treated microglia (Figure 1F,G). iNOS and TNF‐α expression reached a maximum at 8 and 2 h after LPS treatment, respectively (*p* < 0.001; Figure 1F,G). However, no significant difference in Arg‐1 expression was observed at any time points in LPS‐treated microglia (Figure 1H). Third, we examined the effect of IL‐4 on microglia (Figure 1I). Arg‐1 and IGF‐1 mRNA expression showed time‐dependent changes in IL‐4‐treated microglia (Figure 1J,K). The expression of both Arg‐1 and IGF‐1 reached a maximum at 8 h after IL‐4 treatment (*p* < 0.001; Figure 1J,K). In contrast, iNOS mRNA expression showed a time‐dependent decrease in IL‐4‐treated microglia (Figure 1L). iNOS expression reached a minimum at 8 h after IL‐4 treatment (*p* < 0.01; Figure 1L).

### 3.2 LIPUS inhibits LPS‐induced M1 markers and selectively promotes IL‐4‐induced M2 markers

**FIGURE 2:**
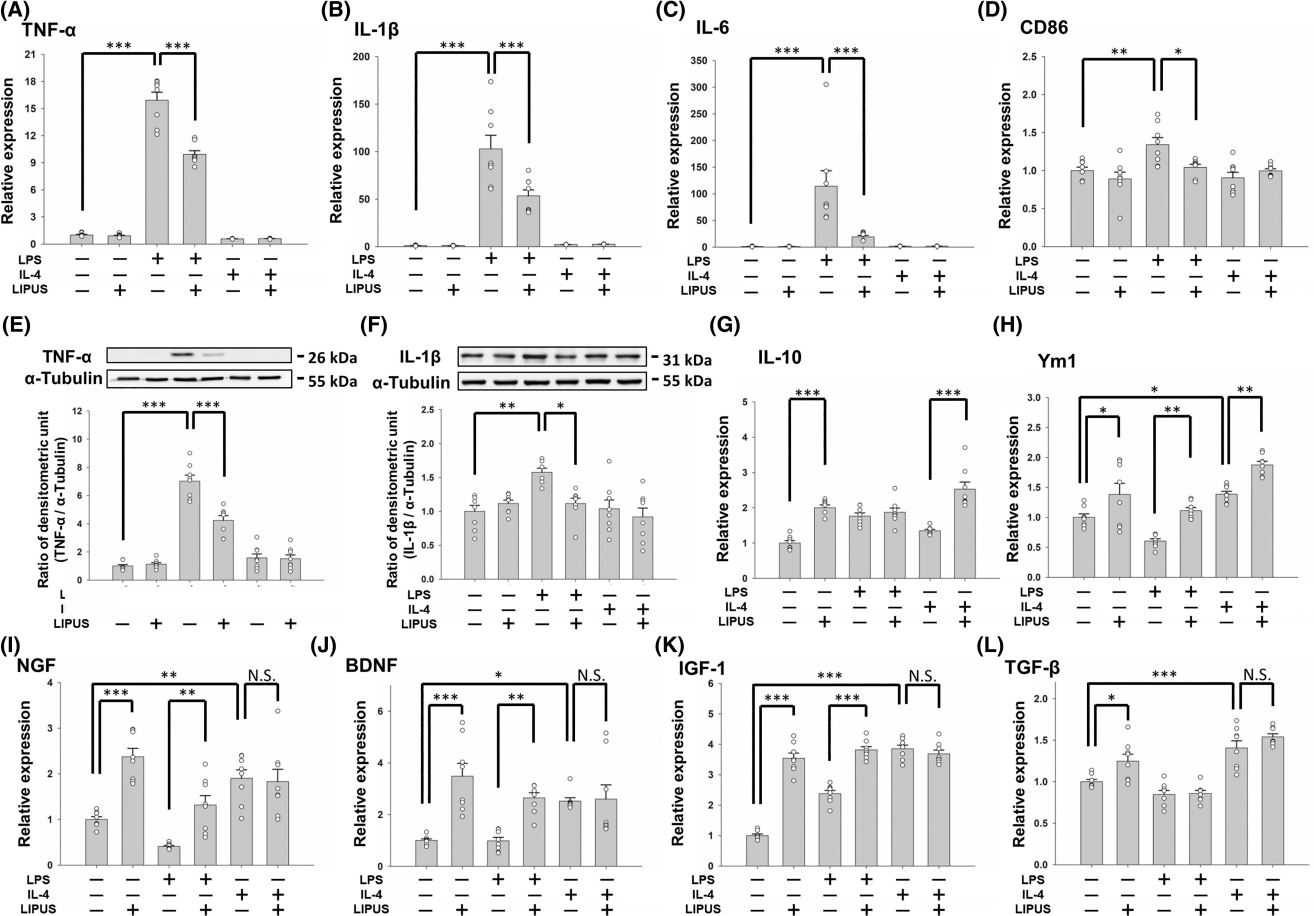
Effects of LIPUS treatment on the expression of M1‐ and M2‐related cytokines and neurotrophins. Microglia were treated with LPS, IL‐4, LIPUS, or a combination of LIPUS and LPS or IL‐4. (A) TNF‐α, (B) IL‐1β, (C) IL‐6, and (D) CD86 mRNA in microglia was quantified by qRT‐PCR. Representative immunoblots and densitometric analyses of (E) TNF‐α and (F) IL‐1β in microglia. (G) IL‐10, (H) Ym1, (I) NGF, (J) BDNF, (K) IGF‐1, and (L) TGF‐β mRNA in microglia was quantified by qRT‐PCR. *p < 0.05; **p < 0.01; ***p < 0.001; N.S. = not significant; *n* = 8.

Based on the temporal expression profile shown in Figure 1, we hypothesized that LIPUS affected M1/M2 polarization. To address this hypothesis, we examined whether M1 marker expression was inhibited by LIPUS treatment in LPS‐treated microglia. The expression of M1‐related markers, including TNF‐α (*p* < 0.001), IL‐1β (*p* < 0.001), IL‐6 (*p* < 0.001), and CD86 (*p* < 0.01), was significantly increased by LPS, but these rising trends were attenuated by LIPUS treatment (Figure 2A–D, third and fourth columns). M1 marker expression was not increased by treatment with IL‐4 (Figure 2A–D, fifth column). Consistent with the mRNA expression, the expression of TNF‐α (*p* < 0.001) and IL‐1β (*p* < 0.01) proteins was also increased by LPS and significantly inhibited by LIPUS (Figure 2E,F). In contrast, the expression of M2 markers (IL‐10 and Ym1) was significantly promoted by LIPUS treatment (Figure 2G,H). The expression of M2‐related markers, including NGF (*p* < 0.01), BDNF (*p* < 0.05), IGF‐1 (*p* < 0.001), and TGF‐β (*p* < 0.001), was significantly increased by IL‐4 treatment, but it was not attenuated by LIPUS treatment (Figure 2I–L, fifth and sixth columns).

### 3.3 LIPUS inhibits LPS‐induced iNOS expression, but not IL‐4‐induced Arg‐1 expression

**FIGURE 3:**
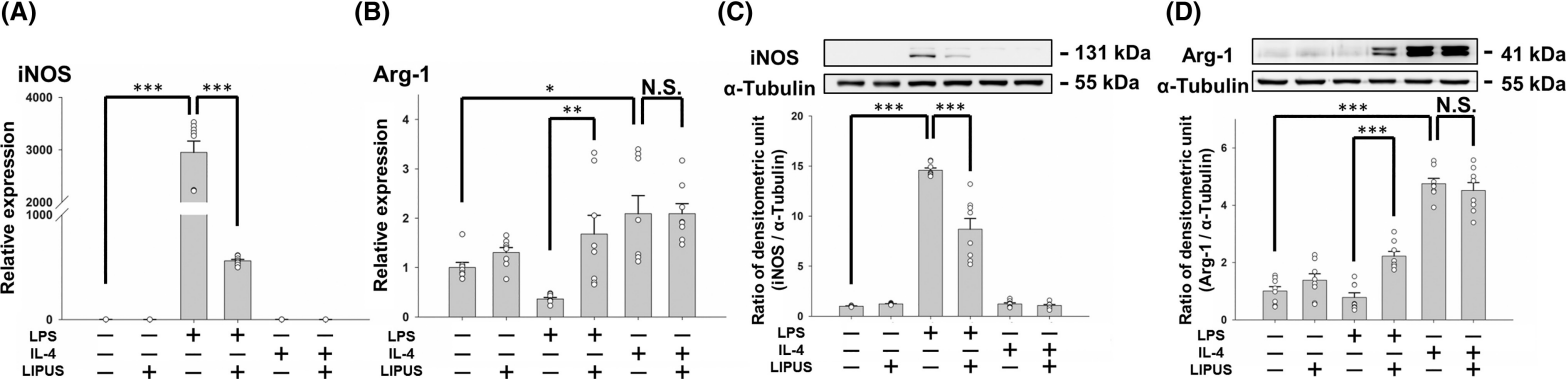
Effects of LIPUS treatment on iNOS and Arg‐1 expression. Microglia were treated with LPS, IL‐4, LIPUS, or a combination of LIPUS and LPS or IL‐4. Representative changes in (A) iNOS and (B) Arg‐1 mRNA were detected by qRT‐PCR at 4 h after treatment with LIPUS. The relative levels of (C) iNOS and (D) Arg‐1 protein were detected by western blotting at 4 h after treatment with LIPUS. *p < 0.05; **p < 0.01; ***p < 0.001; N.S. = not significant; *n* = 8.

The differential expression of iNOS and Arg‐1 in the M1 profile compared with the M2 profile provides a model for microglial phenotypes. LPS treatment increased the mRNA and protein expression of pro‐inflammatory cytokine iNOS (both *p* < 0.001; Figure 3A,C), indicating the shifting of microglia to the M1 phenotype. LPS treatment also decreased the expression of M2‐related Arg‐1 in microglia. LIPUS treatment not only significantly inhibited iNOS mRNA and protein expression in LPS‐treated microglia (both *p* < 0.001; Figure 3A,C), but also significantly promoted Arg‐1 mRNA and protein expression (both *p* < 0.01; Figure 3B,D). However, LIPUS treatment did not induce higher Arg‐1 mRNA and protein expression in IL‐4‐treated microglia (Figure 3B,D). Therefore, these data suggest that LIPUS prevents LPS activation of microglial cells and facilitates their polarization to the M2 phenotype.

### 3.4 LIPUS regulates M1/M2 polarization of microglial cells

**FIGURE 4:**
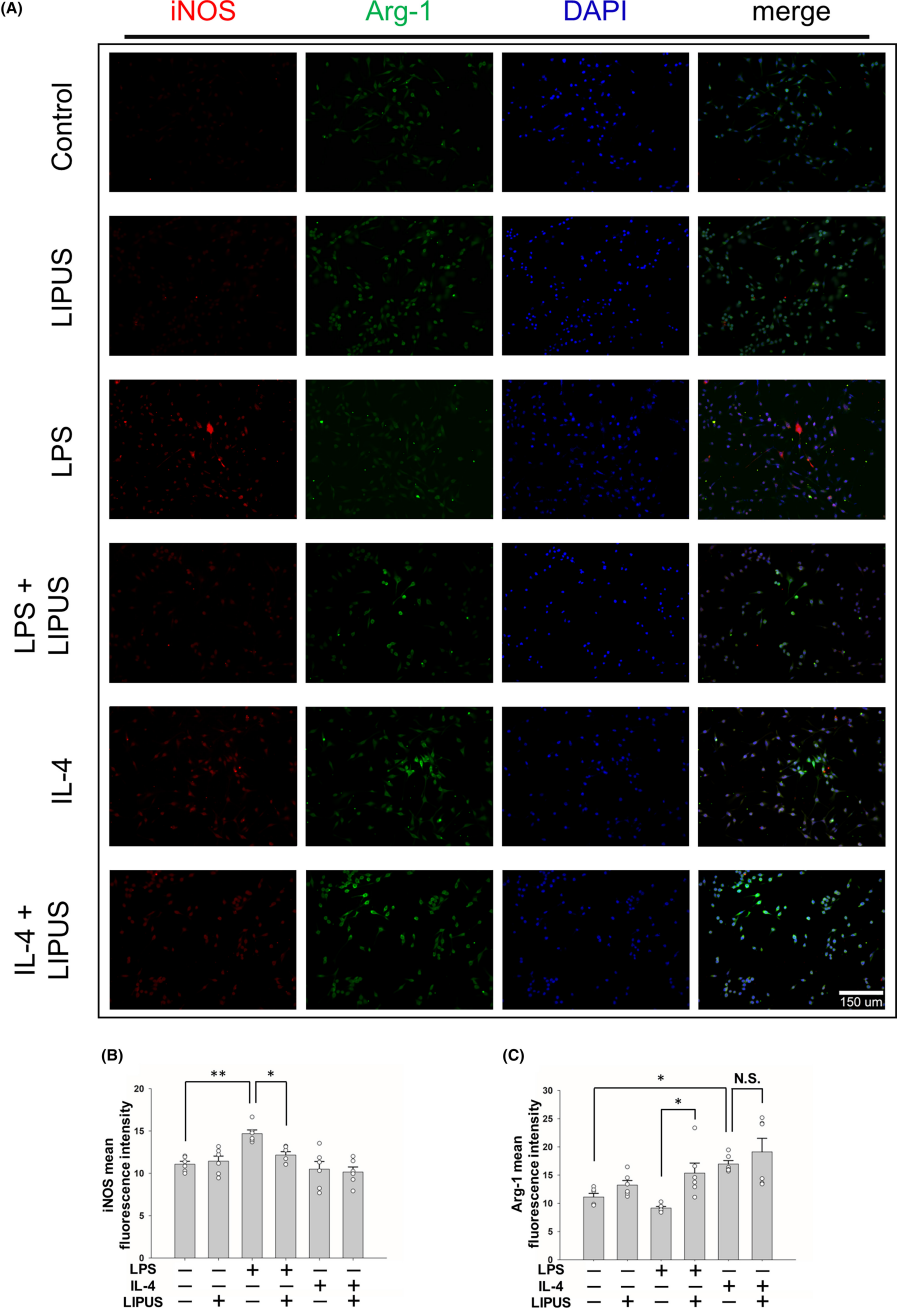
LIPUS treatment regulates M1/M2 microglial polarization. (A) Representative images of immunofluorescent labeling of iNOS (red), Arg‐1 (green), and DAPI (blue) staining of microglia were treated with LPS, IL‐4, LIPUS, or a combination of LIPUS and LPS or IL‐4. The levels of expression of M1 and M2 cell markers (B) iNOS and (C) Arg‐1 were quantified using immunofluorescence at 4 h after treatment with LIPUS. Scale bar = 150 μm. *p < 0.05; **p < 0.01; N.S. = not significant; *n* = 6.

**FIGURE 5:**
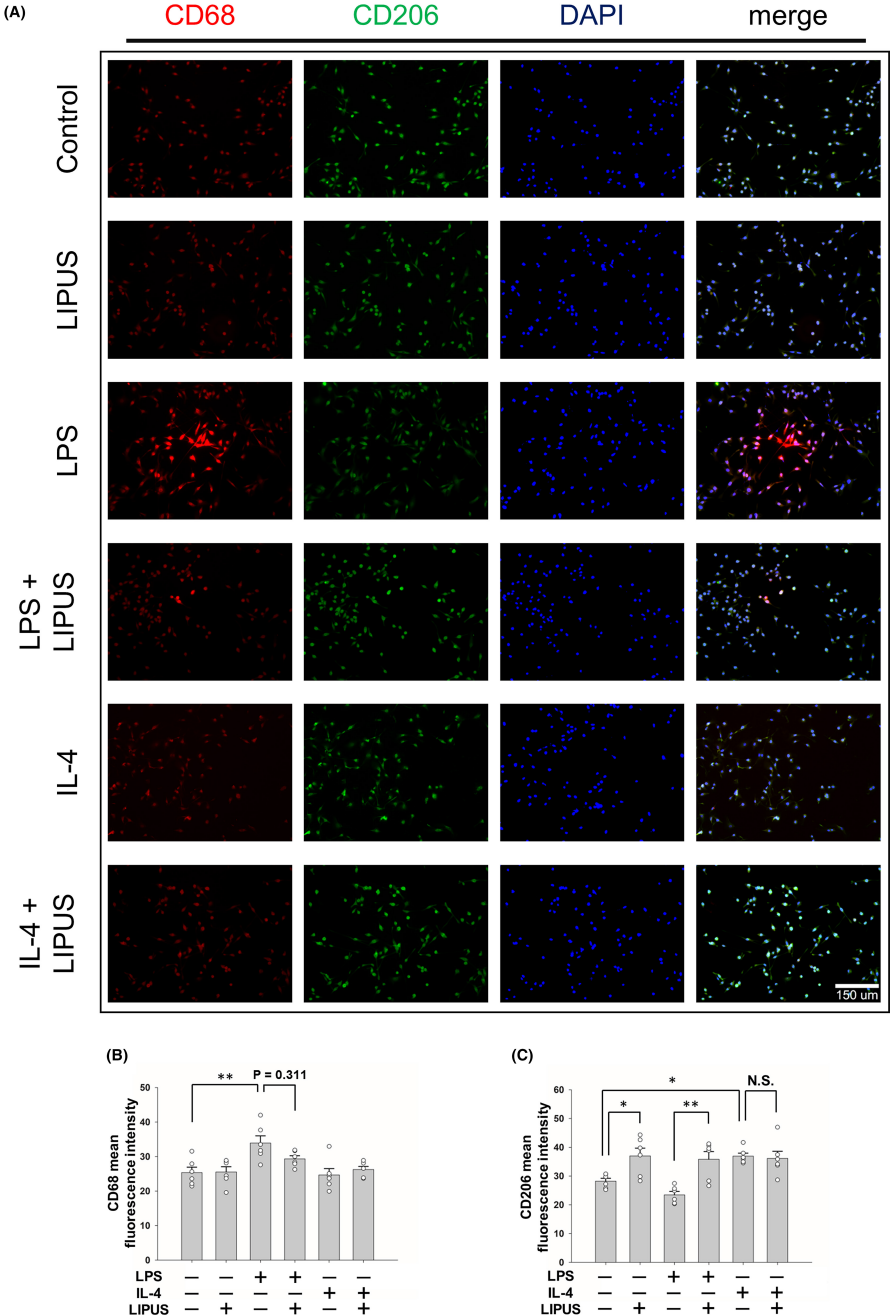
LIPUS treatment regulates M1/M2 microglial polarization. (A) Representative images of immunofluorescent labeling of CD68 (red), CD206 (green), and DAPI (blue) staining of microglia were treated with LPS, IL‐4, LIPUS, or a combination of LIPUS and LPS or IL‐4. The levels of expression of M1 and M2 cell markers (B) CD68 and (C) CD206 were quantified using immunofluorescence at 4 h after treatment with LIPUS. Scale bar = 150 μm. *p < 0.05; **p < 0.01; N.S. = not significant; *n* = 6.

To validate the effect of LIPUS treatment on polarization, microglial cells were stimulated by LPS to an M1 phenotype or by IL‐4 to an M2 phenotype, and then treated with LIPUS. The levels of expression of M1 and M2 intercellular proteins, iNOS and Arg‐1 were detected using immunofluorescence (Figure 4A). Consistent with the mRNA and protein expression shown in Figure 3, iNOS expression was significantly elevated in LPS‐treated microglia (*p* < 0.01; Figure 4B), while it was significantly reduced following LIPUS treatment (*p* < 0.05; Figure 4B, third and fourth columns). In addition, no change in Arg‐1 expression was observed in the microglia treated with LPS, but LIPUS treatment significantly increased Arg‐1 in LPS‐treated microglia (*p* < 0.05; Figure 4C, third and fourth columns). However, no significant difference in Arg‐1 was found in microglia treated with IL‐4 or the combination of IL‐4 and LIPUS (Figure 4C, fifth and sixth columns). The levels of expression of M1 and M2 cell surface markers, CD68 and CD206, were also detected using immunofluorescence (Figure 5A). CD68 expression was significantly elevated in LPS‐treated microglia (*p* < 0.01; Figure 5B), while it was reduced following LIPUS treatment. In addition, no change in CD206 expression was observed in microglia treated with LPS, but LIPUS treatment significantly increased CD206 in LPS‐treated microglia (*p* < 0.01; Figure 5C, third and fourth columns). However, no significant difference in CD206 was found in microglia treated with IL‐4 or the combination of IL‐4 and LIPUS (Figure 5C, fifth and sixth columns). These results suggest that LIPUS reduced M1 polarization of microglia and switched them from M1 to M2 polarization.

### 3.5 LIPUS induces M2 subtype expression in microglia and regulates M1/M2 polarization via the STAT1/STAT6/PPARγ signaling pathways

**FIGURE 6:**
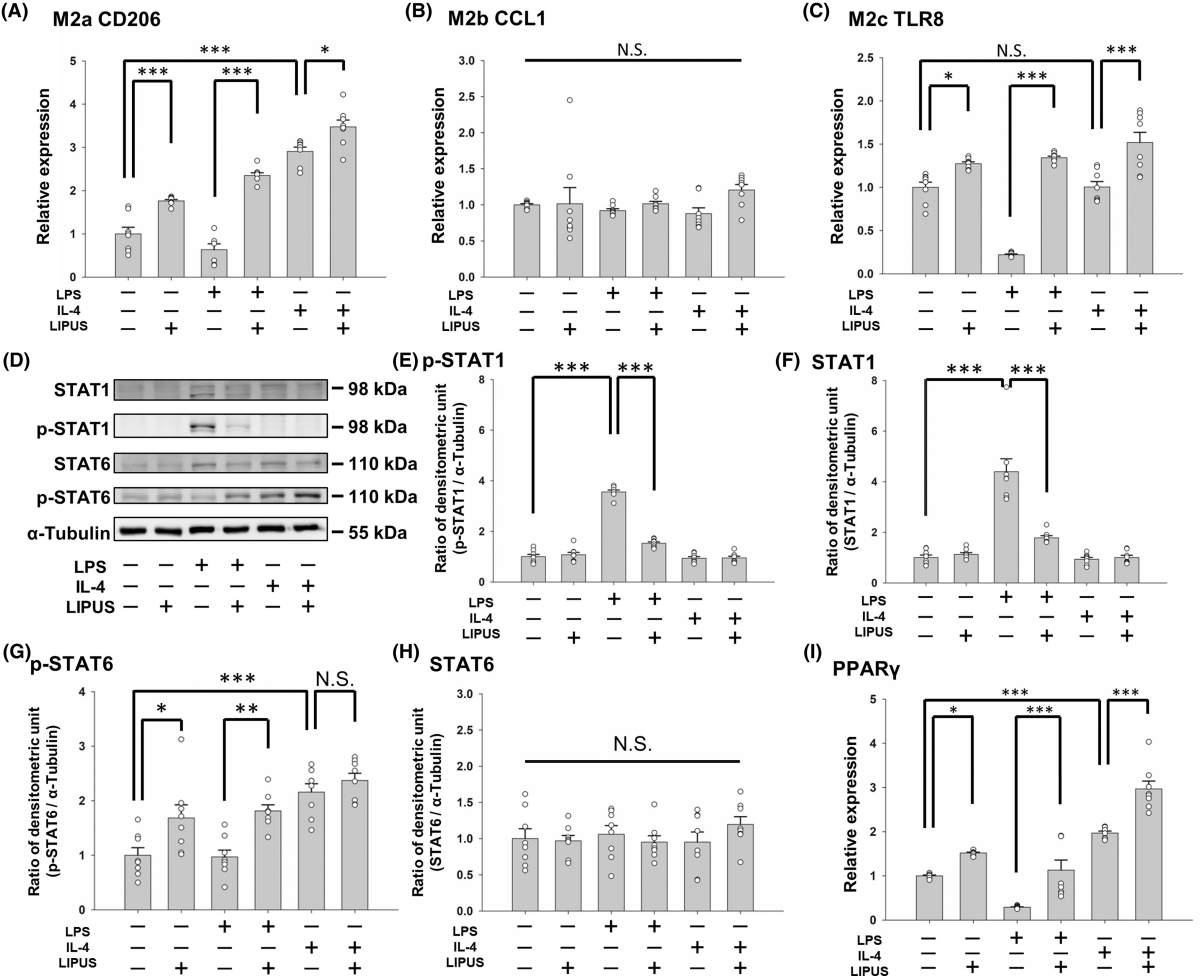
LIPUS induces an M2–mixed phenotype in microglia and modulates the expression of key factors in the STAT1, STAT6, and PPARγ pathways. Microglia were treated with LPS, IL‐4, LIPUS, or a combination of LIPUS and LPS or IL‐4. The mRNA of (A) the M2a marker CD206, (B) the M2b marker CCL1, and (C) the M2c marker TLR8 in microglia was quantified by qRT‐PCR. Representative (D) immunoblots and densitometric analyses of (E) phosphorylated STAT1, (F) STAT1, (G) phosphorylated STAT6, (H) STAT6, and (I) PPARγ using α‐tubulin as an internal control. *p < 0.05; **p < 0.01; ***p < 0.001; N.S. = not significant; *n* = 8.

CD206, CCL1, and TLR8 are markers for microglial subtypes M2a, M2b, and M2c, respectively.^22^ All three M2 phenotypes are anti‐inflammatory repair microglial cells. LIPUS significantly promoted CD206 and TLR8 expression in control, LPS‐treated, and IL‐4‐treated microglia (all *p* < 0.05; Figure 6A, C), while it did not affect CCL1 expression in the three treatments (all *p* > 0.05; Figure 6B). STAT1 and STAT6 have a reciprocal inhibitory relationship and regulate the transcription of M1 or M2 genes involved in the polarization of microglia.^16^, ^25^ To understand the underlying mechanism of the M1/M2 polarization switch after LIPUS treatment, the effects of LIPUS on STAT1/STAT6 were observed. Microglia were treated with LPS to activate the STAT1 pathway, which is necessary for M1 polarization. The level of phosphorylated (p)‐STAT1 protein was significantly increased by LPS and attenuated by LIPUS treatment (Figure 6D,E, third and fourth columns). No change in the level of p‐STAT1 was observed in microglia treated with IL‐4 or the combination of IL‐4 and LIPUS (Figure 6D,E, fifth and sixth columns). We then detected the level of p‐STAT6 protein, which is necessary for M2 polarization. No change in the p‐STAT6 level was observed in microglia treated with LPS, but LIPUS treatment significantly increased p‐STAT6 in LPS‐treated microglia (Figure 6D,G, third and fourth columns). However, no significant effect on p‐STAT6 was found in microglia treated with IL‐4 or the combination of IL‐4 and LIPUS (Figure 6D,G, fifth and sixth columns). To further explore the mechanisms in microglial cells exposed to LIPUS, the expression of PPARγ, a master regulator of M2 polarization of microglia, was measured using real‐time PCR. PPARγ expression was significantly reduced in LPS‐treated microglia compared with control cells (*p* = 0.002; Figure 6I, first and third columns), but this trend was reversed in the presence of LIPUS (*p* < 0.001; Figure 6I, third and fourth columns). Moreover, IL‐4 induced a significant increase in PPARγ mRNA expression compared with control cells; this increase was further enhanced in the presence of LIPUS (both *p* < 0.001; Figure 6I, fifth and sixth columns). Taken together, our results suggest that LIPUS regulates M1/M2 polarization of microglia via the STAT1/STAT6/PPARγ pathways.

## 4 DISCUSSION

Many drugs inducing the M1‐to‐M2 phenotype switch in microglia have therapeutic potential for treating neurodegenerative diseases but have failed due to the limited permeability of the BBB.^26^, ^27^ Therefore, there is clinical interest in changing microglial polarization through innovative approaches without the impediment of the BBB. In vivo and in vitro experiments provide increasing evidence of the anti‐inflammatory effects of LIPUS on LPS‐induced inflammation.^8^, ^9^ LIPUS treatment has been shown to alleviate LPS‐induced neuroinflammation by inhibiting TLR4/NF‐κB signaling and enhancing TrkB/CREB expression in LPS‐treated microglia.^9^ In this study, we demonstrated that noninvasive LIPUS stimulation inhibited M1 polarization and promoted the switch from M1 to M2 polarization in LPS‐treated microglia. Our results suggest further development of LIPUS stimulation as a potential treatment for multiple neurodegenerative diseases.

An initial investigation of time points was performed with LPS, IL‐4, or LIPUS to determine the optimal expressive time points of these three stimulators (Figure 1). To mimic a pro‐inflammatory (M1‐like) or an anti‐inflammatory (M2‐like) classification of microglial activation, we pretreated microglia with LPS or IL‐4, respectively, for 4 h. We then stimulated the microglial cells with LIPUS for 15 min, and then collected the samples. The MTT assay showed that LPS, IL‐4, and LIPUS at these doses did not induce cytotoxicity (Figure 1D). Subsequently, depending on whether the microglial phenotype was M1 or M2 following treatment with LPS, IL‐4, or LIPUS (Figure 3), microglia promoted either iNOS or Arg‐1 to metabolize ʟ‐arginine for the M1 pathogen‐killing response or the M2 wound healing response, respectively.^28^ These results indicate that LIPUS stimulation switched the production of cytotoxic nitric oxide to metabolization of ʟ‐arginine for wound healing in microglia, which is beneficial for alleviating the cytotoxicity induced by microglia in neuroinflammation.^1^, ^2^

The M1 and M2 microglial phenotypes can be converted to each other in specific microenvironments.^29^ Many transcription factors are involved in microglial polarization, for example, STATs, PPARγ, NF‐κB, and CREB, which modulate polarization to a certain phenotype during different inflammatory responses.^30^, ^31^, ^32^, ^33^ Microglia can be polarized to the M1 phenotype or the M2 phenotype through STAT1 or STAT6 activation, respectively.^34^ Our study showed an LPS‐induced increase in M1‐related markers (iNOS, TNF‐α, IL‐1β, and IL‐6) and phosphorylation of STAT1 and an IL‐4‐induced increase in M2‐related markers (Arg‐1, IL‐10, Ym1, and NGF) and phosphorylation of STAT6 (Figures 2, 3, and 6E,G). LIPUS attenuated STAT1 phosphorylation and promoted STAT6 phosphorylation in LPS‐treated microglia, indicating that LIPUS shifts microglia from the M1 to the M2 phenotype via the STAT1/STAT6 signaling pathway (Figure 6E,G). On the other hand, the phosphorylation of both STAT1 and STAT6 did not change after LIPUS treatment of IL‐4‐treated microglia, suggesting that LIPUS did not induce a shift in microglial polarization from M2 to M1.

PPARγ is a widely expressed nuclear transcriptional factor with protective features. It is considered to be a potential treatment target for CNS diseases due to its association with pro‐inflammatory cytokine suppression and inflammatory disease improvement.^35^, ^36^ In a murine model of Alzheimer's disease, PPARγ was found to play a crucial role in the attenuation of inflammation, which may be associated with modulation of M1/M2 polarization.^27^ As shown in Figure 6I, LIPUS treatment significantly enhanced PPARγ activity in control cells and LPS‐treated and IL‐4‐treated microglia. PPARγ activation can promote M2 markers in microglia, such as IL‐10, Arg‐1, and CD206.^31^, ^37^ Arg‐1 is a marker for the M2 phenotype and a known PPARγ target gene. Our results showed that IL‐4 treatment significantly promoted both Arg‐1 mRNA and protein expression in BV2 control cells (Figure 3B,D). However, LIPUS did not induce Arg‐1 upregulation in IL‐4‐treated microglia.

The M2 phenotype can be categorized into M2a, M2b, and M2c subtypes. The M2a subtype is responsible for tissue repair and anti‐inflammatory actions. It is like the M1 subtype in that it releases TNF‐α, IL‐1β, and IL‐6, but it is different from M1 because it upregulates IL‐10 and downregulates iNOS expression. The M2c subtype is involved in inflammation dampening and wound healing. Interestingly, our data showed that LIPUS enhanced the expression of the M2a and M2c markers, CD206 and TLR8, but not the expression of the M2b marker, CCL1 (Figure 6A–C). Therefore, the exact mechanisms underlying LIPUS modulation of PPARγ and the M2 subtypes need further investigation.

## 5 CONCLUSIONS

In summary, we demonstrated that LIPUS inhibited inflammatory responses in activated microglia by shifting them from the M1 phenotype to the M2 phenotype. LIPUS decreased the release of markers of M1 polarization and promoted the expression of markers of M2 polarization by modulating the STAT1/STAT6 and PPARγ signaling pathways. Regulation of the immune response is a promising strategy to prevent neuroinflammation. This study provides new insights into the modulatory mechanisms of microglial polarization by shedding light on potential new therapies based on LIPUS‐mediated microglial polarization for neuroinflammation‐related diseases.

## AUTHOR CONTRIBUTIONS

Chin‐Hung Hsu, Yi‐Ju Pan, and Feng‐Yi Yang designed the study and wrote the manuscript. Chin‐Hung Hsu and Yin‐Ting Zheng performed the experiments and quantification of the data. Feng‐Yi Yang reviewed the manuscript. Chin‐Hung Hsu and Feng‐Yi Yang analyzed the data. Raymond Y. Lo participated in the discussion. All authors read and approved the final manuscript.

## CONFLICT OF INTEREST STATEMENT

The authors declare that there are no conflicts of interest.

## Supporting information


Table S1:

## Data Availability

The data that support the findings of this study are available from the corresponding author upon reasonable request.
