# Supplementary material for: Ultrasound reduces inflammation by modulating M1/M2 polarization of microglia through STAT1/STAT6/PPARγ signaling pathways
Source: CNS Neurosci Ther. 2023 Jul 3;29(12):4113–23. doi: 10.1111/cns.14333 (PMC10651950; doi:10.1111/cns.14333)
Supplement: Supplementary file 1 — Table S1: [file CNS-29-4113-s001.docx]

**Supplementary Information**

**Ultrasound reduces inflammation by modulating M1/M2 polarization of microglia through STAT1/STAT6/PPARγ signaling pathways**

Chin-Hung Hsu^1#^, Yi-Ju Pan^2,3#^, Cong-Yong Gao^1^, Raymond Y. Lo^4^, Feng-Yi Yang^1*^

^1^Department of Biomedical Imaging and Radiological Sciences, National Yang Ming Chiao Tung University, Taipei, Taiwan

^2^Department of Psychiatry, Far Eastern Memorial Hospital, New Taipei City, Taiwan

^3^Department of Chemical Engineering and Materials Science, Yuan Ze University, Taoyuan City, Taiwan

^4^Department of Neurology, Buddhist Tzu Chi General Hospital and Tzu Chi University, Hualien, Taiwan

^#^These authors contributed equally to this work.

*Correspondence to:

Feng-Yi Yang, Ph.D.

Professor, Department of Biomedical Imaging and Radiological Sciences, School of Biomedical Science and Engineering, National Yang Ming Chiao Tung University, Taipei, Taiwan

No. 155, Sec. 2, Li-Nong St., Taipei 11221, Taiwan

Tel: 886-2-2826-7281, Fax: 886-2-2820-1095

E-mail: fyyang1@nycu.edu.tw

**Running title:** Ultrasound modulates microglia

**Table S1: Primers used for qRT-PCR.**

| **Gene** | **Sense(5’-3’)** | **Anti-sense(5’-3’)** |
| --- | --- | --- |
| β-actin | CATTGCTGACAGGATGCAGAAGG | TGCTGGAAGGTGGACAGTGAGG |
| IL-1β | GGAGAACCAAGCAACGACAAAATA | TGGGGAACTCTGCAGACTCAAAC |
| IL-6 | TAGTCCTTCCTACCCCAATTTCC | TTGGTCCTTAGCCACTCCTTC |
| TNF-α | CCTCCCTCTCATCAGTTCTA | ACTTGGTGGTTTGCTACGAC |
| iNOS | GTTCTCAGCCCAACAATACAAA | GTGGACGGGTCGATGTCAC |
| CD68 | ATTGAGGAAGGAACTGGTGTAG | CCTCTGTTCCTTGGGCTATAAG |
| Arg1 | GAATCCCACCTAGGAGACAAAG | GTCTATACTCCCTGCAGTTTCC |
| CD206 | TGAGCTGTTTTGGTTGGGAC | CCCATCTGCAGTAACTGGTG |
| IL-10 | TTGAATTCCCTGGGTGAGAAG | TCCACTGCCTTGCTCTTATTT |
| BDNF | CTGAGCGTGTGTGACAGTATTA | CTTTGGATACCGGGACTTTCTC |
| NGF | CAGTGAGGTGCATAGCGTAAT | CTCCTTCTGGGACATTGCTATC |
| TGF-β | CCAGATCCTGTCCAAACTAAGG | CTCTTTAGCATAGTAGTCCGCT |
| Ym1 | TGAATGAAGGAGCCACTGAG | TTGTTGTCCTTGAGCCACTG |
| PPARγ | CCCTGGCAAAGCATTTGTAT | CACCTCTTTGCTCTGCTCCT |
| CCL1 | GGATGTTGACAGCAAGAGCA | TAGTTGAGGCGCAGCTTTCT |
| TLR8 | ACAATGCTCCATTTCCTTGC | CTGAGGGAAGTGCTGGAAAG |
